# Development-associated immunophenotypes reveal the heterogeneous and individualized early responses of adult B-acute lymphoblastic leukemia

**DOI:** 10.1097/MD.0000000000004128

**Published:** 2016-08-26

**Authors:** Hui-Fang Li, Wen-Tong Meng, Yong-Qian Jia, Neng-Gang Jiang, Ting-Ting Zeng, Yong-Mei Jin, Qiao-Rong Huang, Xue Li, Hong Xu, Xian-Ming Mo

**Affiliations:** a Laboratory of Stem Cell Biology, State Key Laboratory of Biotherapy; b Department of Hematology; c Department of Laboratory Medicine, West China Hospital; d Collaborative Innovation Center for Biotherapy, Sichuan University, Chengdu, China.

**Keywords:** adult B-acute lymphoblastic leukemia, early responses, leukemia cell subpopulations, multicolor flow cytometry

## Abstract

B cell acute lymphoblastic leukemia (B-ALL) exhibits phenotypes reminiscent of normal stages of B-cell development. As demonstrated by flow cytometry, the immunophenotypes are able to determine the stages of B cell development. Multicolor flow cytometry (MFC) is more accurate at identifying cell populations. In this study, 9-color panels, including CD10, CD19, CD20, CD22, CD34, CD79a, CD179a, and IgM, which are sequentially expressed during B cell development, were designed to detect the leukemia cell subpopulations in adult B-ALL patients. In 23 patients at diagnosis, 192 heterogeneous subpopulations of leukemia cells were detected. Compared with their counterparts at diagnosis and after the 1st course of induction therapy, the responses of the subpopulations were also heterogeneous. In the CD10^+^ population, the residual B cell subpopulations in the BCR/ABL^+^ patients were obviously reduced compared to those in the BCR/ABL^−^ patients. New subpopulations were detected in 22 of 23 patients and were primarily located in the CD34^+^CD10^−^ populations. Subpopulations of clonal evolution were heterogeneous after induction therapy. Our results suggest that the subpopulations in B-ALL patients should be dynamically monitored by development-associated immunophenotyping before, during, and after induction therapy and to predict the prognosis of the disease.

## Abstract 2

Supplemental Digital Content is available in the text

## 1 Introduction

B cell acute lymphoblastic leukemia (B-ALL) is a clonal, malignant disease that originates from a single cell and is characterized by the accumulation of blast cells that are phenocopies of the B-cell developmental stages.^[1]^ In childhood B-ALL, the leukemia cell often displays significant heterogeneity in its morphology, immunophenotype, genetic aberrations (Ig/TCR gene rearrangement), and therapeutic response.^[2,3]^ The presence of coexisting subclones in B-ALL has been well reported. Approximately 99% of the subclones are present at a frequency of less than 0.1% at diagnosis.^[4]^ Immunophenotypically heterogeneous leukemia cell populations are distinct subpopulations with bimodal or broad expression of surface markers in childhood B-ALL. We have also found that the immunophenotypic patterns of 51 common adult B-ALL are highly heterogeneous.^[5]^ Mullighan^[6]^ revealed that 52% of the relapsed ALL clones are derived from minor “ancestral” subclones that are present at diagnosis. The data suggest that the success of the treatment of most ALL patients should not be measured by the loss of the predominant clone at diagnosis, but rather by the effects on numerous underappreciated subclones. Subpopulations of B-ALL cells are relevant for understanding the ontogeny of the malignant cells and are able to provide clues for understanding the biological mechanisms of therapeutic resistance and relapse.^[3]^

Flow cytometry (FCM) or PCR are currently used to determine the subpopulations of B-ALL cells. PCR has a higher sensitivity (10^−5^–10^−6^) than FCM (10^−4^).^[7–9]^ FCM and PCR cannot simply substitute for each other. The concordance rates between their results depend largely on the time at which they are used.^[10]^ Currently, multicolor flow cytometry (MFC) is able to acquire more cells, has a much higher resolution and capacity for detecting rare ALL subclones, and is reliable for monitoring subpopulations related to minimal residual disease in B-ALL.^[11]^ Moreover, the specific advantages of FCM include the potential for analyzing the status of normal hematopoietic cells, while searching for subpopulations and obtaining information about the degree of lympho-hematopoietic recovery during and after the therapy. MFC has become the preferred method to assess the immunophenotypic features of cells present in the peripheral blood, bone marrow (BM), lymph node biopsy specimens, and other types of samples that are suspected of containing oplastic hematopoietic cells.^[12–14]^ Accordingly, EuroFlow provides comprehensive 8-color panels aimed at standardizing the procedure for the immunophenotypic diagnosis and classification of B-ALL.^[15,16]^

The differentiation of B cells from early committed progenitors into mature B-lymphocytes is a multistep maturation process.^[1]^ The sequential stages of B cell development have been well established, hematopoietic stem cell → common lymphoid precursor (CLP) → early-B → pro-B → pre-B → immature-B → mature-B, by monitoring the levels of surface and intracellular markers.^[1,17]^ The correlation between the normal and leukemic B-cells has been determined by the combined evaluation of their microscopic appearance and immunophenotypes.^[1,18]^ Childhood ALLs include phenotypically distinct B-cell stages, including Pro-B-like (CD34^+^CD38^+^CD19^+^) and CD34^+^CD38^−/low^CD19^+^cells dubbed “Stem/B” cell, which are only observed in leukemia and preleukemia. BCR/ABL1^+“^Stem/B” cells that selectively persist at remission are more quiescent (G0) and cycle less actively (S–M–G2) than leukemic “Pro-B” cells.^[19]^ The speed of blast clearance during therapy is a major prognostic factor of the outcome in childhood ALL.^[9,20–22]^ The blast counts in the BM on days 15 and 33 have been widely used to deliver risk-directed therapy.^[21]^

To estimate the reductions in the early leukemia cell subpopulations’ parameters, we investigated the changes in the lymphocytoid subpopulations in the BM of adult B-ALL patients at diagnosis and after the 1st course of induction therapy. In this analysis, we analyzed the low and insensitive lymphocytoid cell subpopulations after induction therapy using MFC. We found that adult B-ALL patients often displayed a massive collection of subtly divergent leukemic subclones. The responses of leukemia cell subpopulations to induction therapy were individualized, and the subpopulations of evolved clones were also heterogeneous.

## 2 Material and methods

### 2.1 Patients and samples

Twenty-three adult B-ALL patients who were 1st diagnosed at the Western China Hospital were enrolled in this study (Table 1). These patients had been given a definitive diagnosis of B-ALL in accordance with the World Health Organization classification (2008). The symptoms and data from physical examinations and some pathologic examinations were collected from the 23 patients (Table 1). For the early response, the subpopulations were evaluated in follow-up BM samples from the 23 cases that were obtained before and after the 1st course of induction therapy, which was based on vincristine, prednisone, and/or the addition of anthracycline, asparaginase, or both. In addition, patients positive for the BCR-ABL fusion gene were administered Imatinib. Besides that, there are many other kinds of medicines used shown in the Supplementary Table 1. Moreover, patients with other malignant diseases, such as MDS and CML, were excluded. The study was conducted according to the requirements of the institutional ethical committee, and informed consent was obtained from each patient.

**Table 1:**
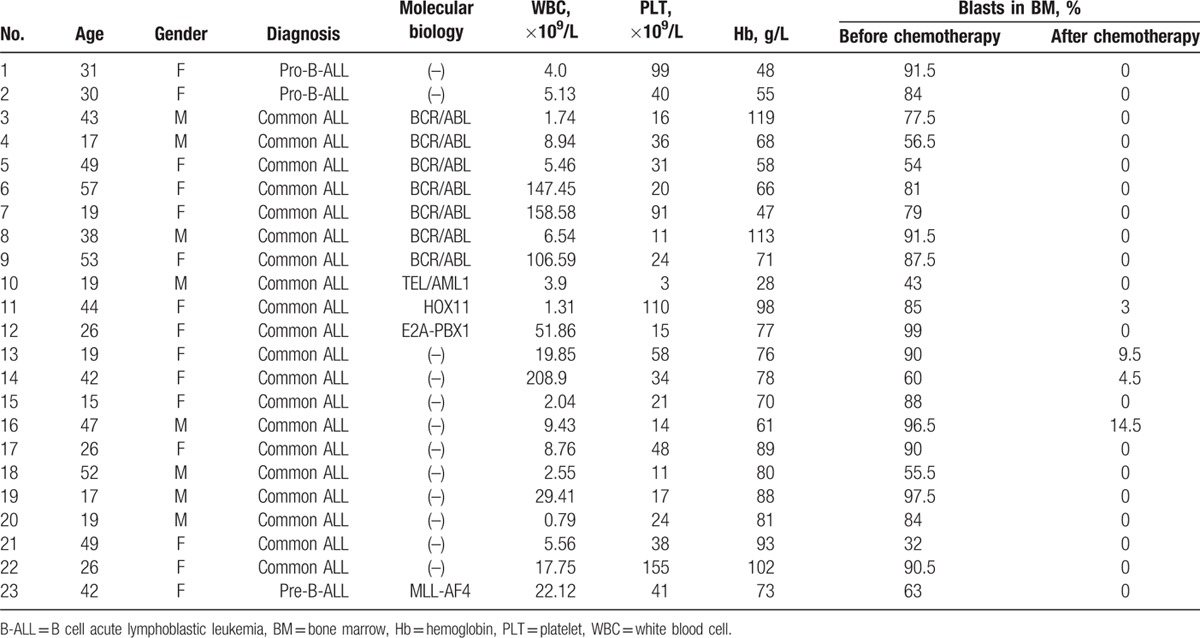
The basic characteristics of the 23 B-ALL patients.

### 2.2 FCM assays

A 9-color combination of V450/V500/FITC/PE/PE-CF594/PerCP-Cy5.5/PE-Cy7/APC/APC-Cy7 was used for the assay (Table 2). The panel included CD19/CD45/sIgM/cCD179a/CD20/CD22/CD34/cCD79a/CD10. The following antibodies were used: CD10 from BioLegend (San Diego, CA); CD179a from AbD Serotec (Kidlington, UK); and all others were from Becton Dickinson (BD, San Jose, CA). Each antibody was titrated by serial dilutions. BM samples containing up to 3 × 10^6^ WBCs were lysed with an ammonium chloride solution. Then, the cell suspensions were centrifuged for 5 minutes at 600 g. The cell pellet was resuspended in PBS and washed twice by centrifugation at 600 g for 5 minutes. The cell pellet was then resuspended in 50 μL of PBS and incubated with the surface marker antibodies (CD10, CD19, CD20, CD22, CD34, sIgM, and CD45) for 30 minutes at room temperature in the dark. Then, the cells were fixed and permeabilized using Fixation and Permeabilization reagent (BD) and incubated for 10 minutes at room temperature. Subsequently, the CD79a and CD179a antibodies were added and incubated for 30 minutes at room temperature in the dark, followed by 2 washes with centrifugation before acquisition.

**Table 2:**
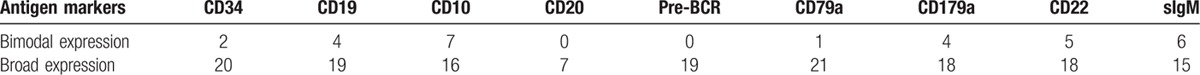
Antigen expression model in 23 B cell acute lymphoblastic leukemia (B-ALL) patients at diagnosis.

### 2.3 Data acquisition and analysis

Data acquisition and analysis were performed on a FACSAria cytometer equipped with the DivaV6.0 software (Becton Dickinson, San Jose, CA). The instrument was standardized to reduce batch-to-batch shifting by daily monitoring with Rainbow beads (BD). The boundary between the positive and negative cells was set using fluorescence-minus-one controls and an internal control. The maximum possible number of cells was acquired (at least 500,000 events and preferably 1 million or more events). The normal cells present in the BM were used as positive and negative controls for the antibody performance. A sequential gating strategy was utilized in the analysis (Supplementary Figure 1a). The aggregated cells were excluded based on the forward scatter (FSC) height versus FSC area dot plots. The dead cells and debris were excluded from the FSC/side scatter (SSC) dot plots. The blast populations were identified with CD34/CD19 dot plots after being analyzed by the SSC/CD45 dot plots. By backgating into the SSC/CD45 plots, we verified that minor subpopulations were always located in the region where the blasts were normally found. At least 1 cell of a particularly phenotype was required to be considered a cell subpopulation. A chip-graph created with Origin 9.0 software (OriginLab, CA) was used to analyze the data.

The blast populations were identified using the CD34/CD19 dot plots. The CD34^−^CD19^−^ population was excluded because they contained cell debris and erythroblasts, and the other 3 main populations were further gated by the expression of the other antigens (Supplementary Figure 1a). Eight antigens associated with B-lymphoid development were designed. According to the normal development stages of B lymphocytes, the blasts were divided into 192 subpopulations (Supplementary Figure 1b). We established CD34, CD10, and CD19 as the backbone and took the expression of the other 5 antigens into account. Seven subpopulations, which were consistent with the normal development stages, could be defined: the CLP-like subpopulation (CD34^+^CD10^+^CD79a^−^CD179a^−^CD19^−^CD22^−^CD20^−^IgM^−^), early B-like subpopulation (CD34^+^CD10^+^CD79a^+^CD179a^+^CD19^−^CD22^−^CD20^−^IgM^−^), pro-B-like subpopulation (CD34^+^CD10^+^CD79a^+^CD179a^+^CD19^+^CD22^+^CD20^−^IgM^−^), pre-B1/B2-like subpopulation (CD34^−^CD10^+^CD79a^+^CD179a^+^CD19^+^CD22^+^CD20^+^IgM^−^), pre-B3-like subpopulation (CD34^−^CD10^+^CD79a^+^CD179a^−^CD19^+^CD22^+^CD20^+^IgM^−^), immature B-like subpopulation (CD34^−^CD10^+^CD79a^+^CD179a^−^CD19^+^CD22^+^CD20^+^IgM^+^), and mature B-like subpopulation (CD34^−^CD10^−^CD79a^+^CD179a^−^CD19^+^CD22^+^CD20^+^ IgM^+^).

**Figure 1:**
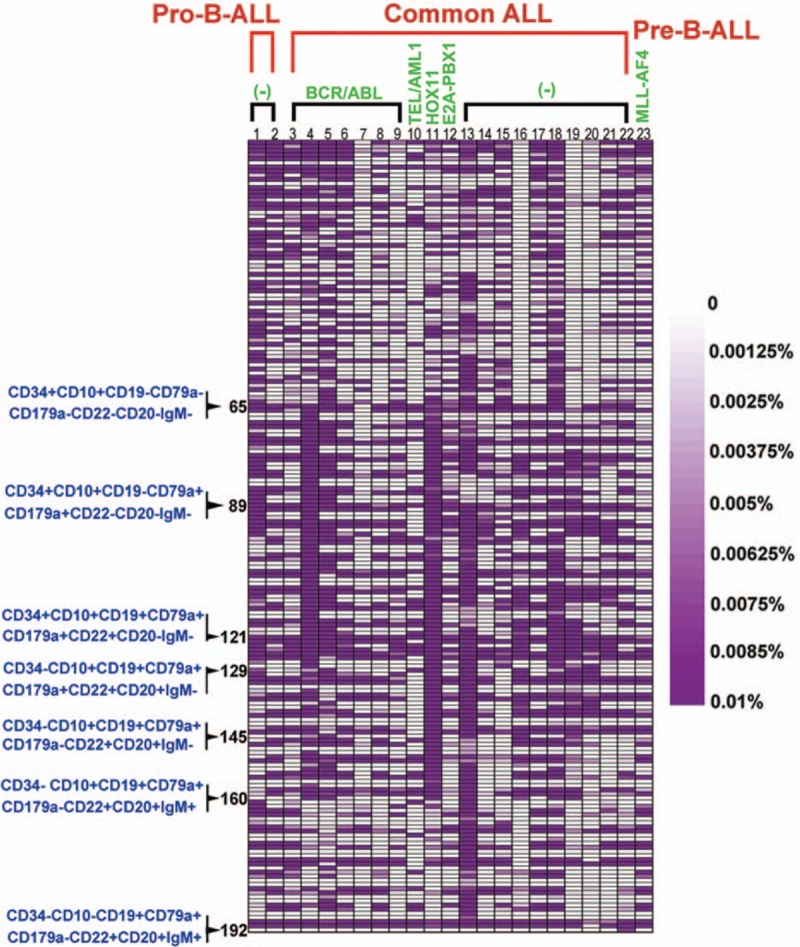
The distribution of the leukemia cell subpopulations at diagnosis. The figure shows the distribution of the leukemia cell subpopulation at diagnosis. The range of 0% to 0.01% (0–100 cells) was defined, and with increasing numbers of cells, the purple color deepened, at the maximum of 0.01% (100 cells), the purple color was the deepest. We established CD34, CD10, and CD19 as the backbone, and took the expression of the other 5 antigens into account. Seven subpopulations, which were consistent with the normal development stages, could be defined. The 65th subpopulation represents the CLP-like subpopulation, the 89th subpopulation represents the early B-like subpopulation, the 121st subpopulation represents the Pro-B-like subpopulation, the 129th subpopulation represents the Pre-B1/B2-like subpopulation, the 145th subpopulation represents Pre-B3-like subpopulation, the 160th subpopulation represents the immature B-like subpopulation, and the 192nd subpopulation represents the mature B-like cell subpopulation.

**Figure 2:**
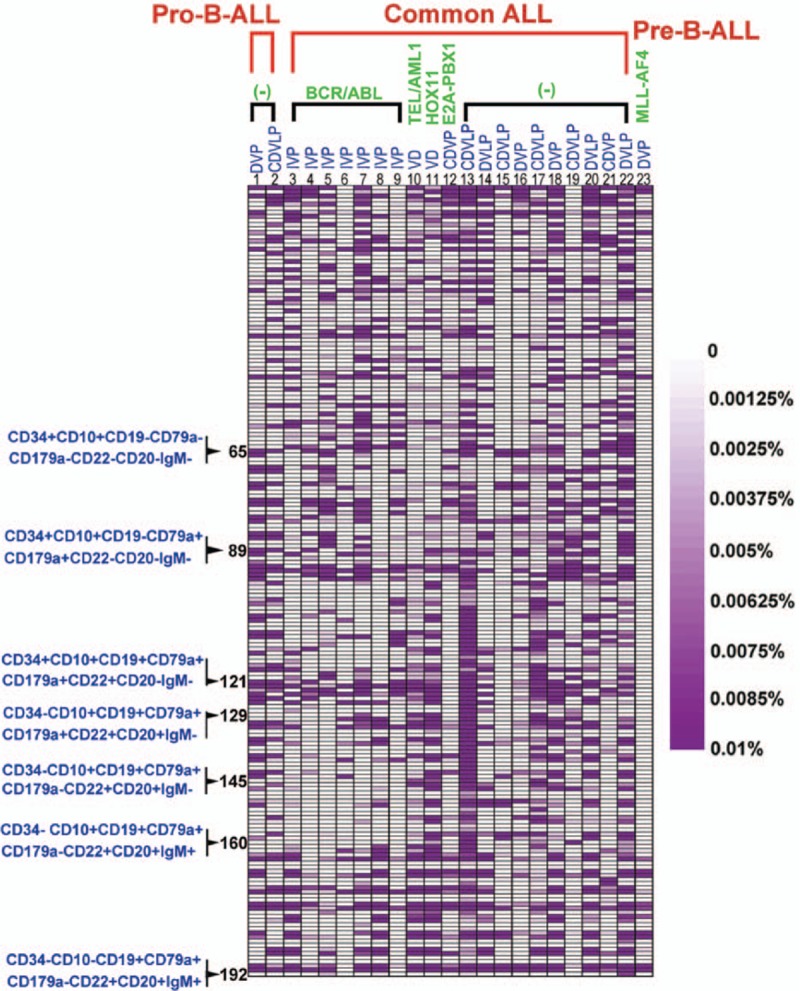
The distribution of the residual lymphoid cell subpopulations. The figure shows the distribution of the residual lymphoid cell subpopulations after induction therapy. The range of 0% to 0.01% (0–100 cells) was defined, and with increasing numbers of cells, the purple color deepened more and reached the maximum of 0.01% (100 cells), when the purple color was the deepest.

**Figure 3:**
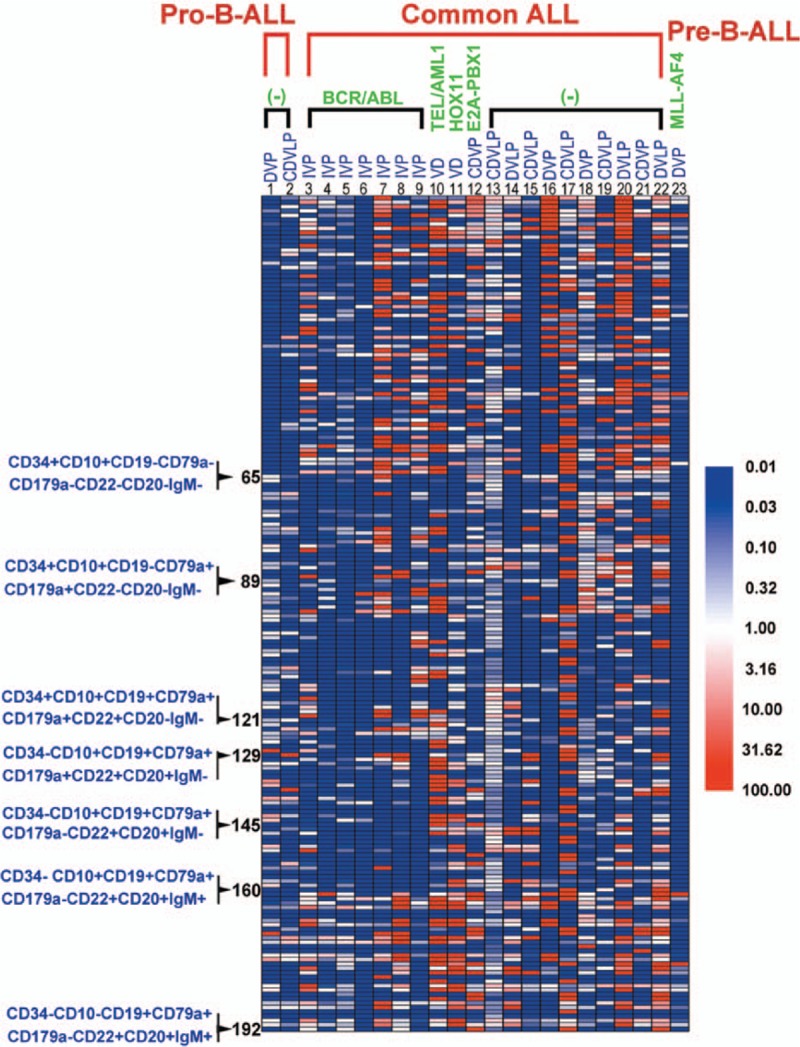
New subpopulations were identified after induction chemotherapy. The figure represents the new subpopulations that were identified after induction chemotherapy. The range of 0% to 0.01% (0–100 cells) was defined, and with increasing numbers of cells, the purple color deepened more and reached the maximum of 0.01% (100 cells), where the purple was the deepest. (−) = BCR/ABL1 fusion gene negative, C = cyclophosvnamide, D = daunorubicin, I = Imatinib, L = asparaginase, P = prednisone/dexamethasone, V = vincristine.

**Figure 4:**
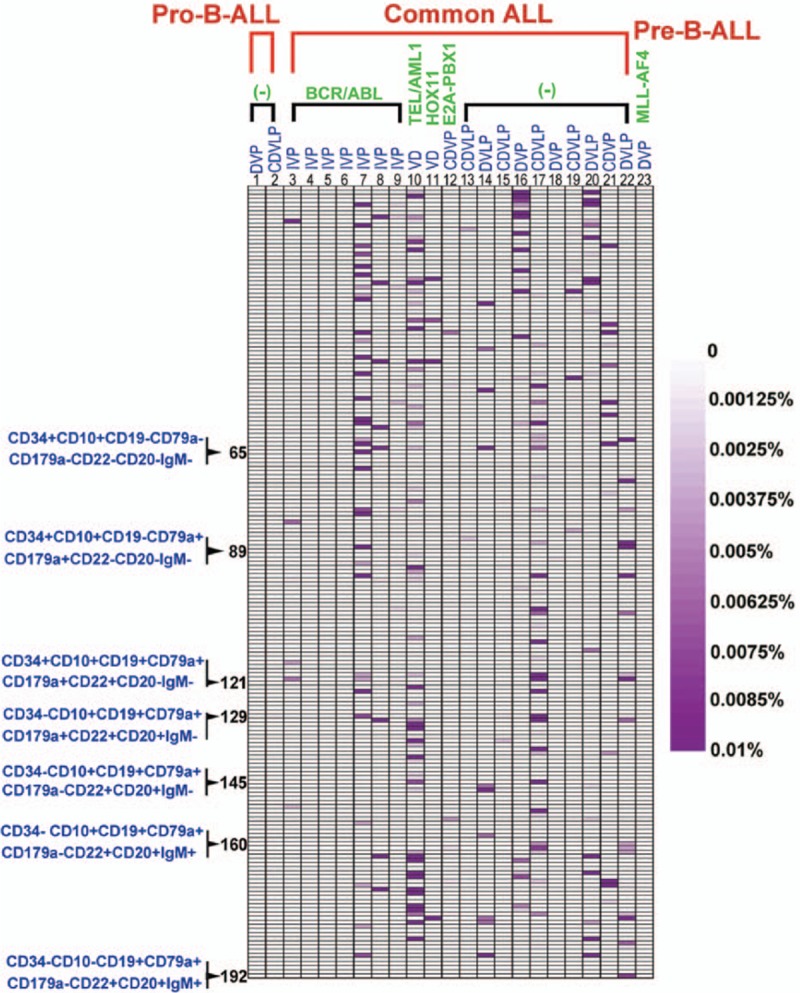
The changes in the same subpopulation between diagnosis and after induction therapy. The figure represents the changes in the same subpopulation between diagnosis and after induction chemotherapy. To compare the changes in the same subpopulation in different B-ALL patients at diagnosis, after induction chemotherapy and at relapse, we used the following formula: RPSs before and after chemotherapy = the percentage of a subpopulation after induction chemotherapy or at relapse/the percentage of a subpopulation at diagnosis. Compared to the samples obtained at diagnosis, we could characterize the changes in the subpopulations as stable, increased or decreased after induction therapy or at relapse. The RPS values were presented as a bicolor chip-graph: the white grid represented the same percentage compared to the samples at diagnosis (RPS = 1), and the subpopulation is defined a subpopulation with a stable or increased percentage when RPS is equal or more than 1; the red grid represented an increased percentage of subpopulations after induction chemotherapy compared to that at diagnosis (RPS > 1). The extent of the increase was represented by the depth of the red color. It peaked when the ratio of the percentage of a subpopulation after induction chemotherapy to the percentage of a subpopulation at diagnosis reached the maximum of 100 (RPS = 100) (including the new subpopulations that were not present at diagnosis, but were identified after induction therapy). Similarly, the blue grid represented the decreased percentages of subpopulations after induction chemotherapy compared to those at diagnosis. The depth of the blue color represented the extent of the decrease. It peaked when the ratio of the percentage of a subpopulation after induction chemotherapy to the percentage of subpopulation at diagnosis reached the minimum of 0.01 (RPS = 0.01, including leukemia cell subpopulations that were present at diagnosis and were not identified after induction therapy and those that were not present at diagnosis or after induction therapy too). (−) = BCR/ABL1 fusion gene negative; C = cyclophosvnamide, D = daunorubicin, I = Imatinib, L = asparaginase, P = prednisone, RPS = ratios to percentages of subpopulation, V = vincristine.

Distinct subpopulations were defined as separate populations, with each having their own peak in the contour plots (In FlowJo; resolution: 128, percentage: 10) and histograms (described as bimodal expression). We defined a marker as broadly expressed when a population had only 1 peak, using the outline of the 10% contour plot as the boundary that extended from 1 score into the middle of the neighboring score.^[3,5]^ The percentage of every subpopulation could be calculated as the number of cells in every subpopulation divided by the number of nucleated cells (Supplementary Figure 1b). In Figs. 1–3, we defined the range of 0% to 0.01% (0–100 cells), and with an increasing number of cells, the purple color deepened more and reached the 0.01% maxima (100 cells), where the purple color was the deepest. To compare the changes in the same subpopulation in different B-ALL patients between diagnosis and after induction chemotherapy, we used the following formula: ratios to percentages of subpopulations (RPS) before and after chemotherapy = the percentage of a subpopulation after induction chemotherapy or at relapse/the percentage of a subpopulation at diagnosis. Compared to the subpopulation at diagnosis, we could determine whether the changes were stable, increased, or decreased after induction therapy. The subpopulation is defined a subpopulation with a stable or increased percentage when RPS is equal or more than 1 (Fig. 4).

### 2.4 Statistical methods

Complete remission (CR) was defined by <5% blast cells in a regenerated BM aspirate, a lack of extramedullary leukemia and platelet and neutrophil counts of >100 × 10^9^/L and 1.5 × 10^9^/L, respectively.^[23]^ The numbers of lymphoid cell subpopulations and changes in the percentages of subpopulations are represented by the mean, median, maximum, and minimum. The rank sum test was used to determine the relationship between the changes in the percentages of subpopulations and the different types of B-ALL patients. *P*-values <0.05 were considered statistically significant. All of these statistical procedures were performed with SPSS, version 16.0 (IBM, NY).

## 3 Result

### 3.1 The heterogeneity of the leukemia cell subpopulations in adult B-ALL patients at diagnosis

We measured the leukemia cells in 23 patients with B-ALL using 9-color MFC. The results showed that the distribution of 192 leukemia cell subpopulations was variable in every patient (Fig. 1). The subpopulations appeared to have a lower tendency to accumulate and presented a significant individual characteristic (significant inter-individual variation). Overall, these leukemia cell subpopulations were characterized by bimodal expression, and the broad expression of antigens was associated with B cell development (Table 2). Many leukemia cell subpopulations were highly similar to the B cell populations that appeared during the developmental program. Additional cell subpopulations were also revealed (Fig. 1). The CD34^+^CD10^−^ populations were identified and primarily expressed one of the antigens associated with the B-lymphoid lineage. Among the samples, 7 patients expressed CD20 and no additional antigens. Sixteen patients carried many lymphoid cell subpopulations that expressed CD20 with additional antigens. For instance, 4 patients displayed CD79a^+^CD20^+^CD19^−^CD179a^−^CD22^−^IgM^−^ subpopulations. Six patients displayed CD179a^+^CD20^+^CD19^−^CD179a^−^CD22^−^IgM^−^ subpopulations. Six patients carried IgM^+^CD20^+^CD19^−^CD79a^−^CD179a^−^CD22^−^ subpopulations. Six patients displayed CD19^+^CD20^+^CD79a^−^CD179a^−^CD22^−^IgM^−^ subpopulations, and 5 patients displayed CD19^+^IgM^+^CD20^+^CD79a^−^CD179a^−^CD22^−^ subpopulations. Three patients displayed CD19^+^CD79a^+^CD20^+^CD179a^−^CD22^−^IgM^−^ subpopulations. Nine patients carried the CD19^+^CD22^+^IgM^+^CD20^+^CD79a^−^CD179a^−^ subpopulations. Of the CD34^+^CD10^+^ and CD34^−^CD10^+^CD19^+^CD179a^+^ populations, the leukemia cell subpopulations exhibited a central tendency. Twenty-three patients carried the CD34^+^CD10^+^CD79a^+^CD179a^+^CD19^+^CD22^+^CD20^−^IgM^−^ and CD34^+^CD10^+^CD79a^+^CD179a^+^CD19^+^CD22^−^CD20^−^IgM^−^ subpopulations. Twenty-two patients carried the CD34^+^CD10^+^CD79a^+^CD179a^+^CD19^+^CD22^+^CD20^−^IgM^+^ and CD34^+^CD10^+^CD79a^+^CD179a^+^CD19^+^CD22^−^CD20^−^IgM^+^ subpopulations. Four patients displayed the CD34^+^CD10^+^CD79a^−^CD179a^−^CD19^+^CD22^−^CD20^+^IgM^−^ subpopulation. Seven patients displayed the CD34^+^CD10^+^CD79a^−^CD179a^−^CD19^+^ CD22^−^CD20^+^IgM^+^ subpopulation. The distribution of the leukemia cell subpopulations in the samples from patients who expressed BCR/ABL showed little difference to the samples from patients who did not express BCR/ABL. Most of the CD34^−^CD10^+^CD19^+^CD179a^−^ and CD34^−^CD10^−^CD19^+^ leukemia cell subpopulations did not express CD20. The leukemia cell subpopulations were more centralized in the pre-B-ALL patients. The CD34^−^CD10^−^CD19^+^ leukemia cell subpopulations in the pro-B-ALL patients were significantly less compared to the common ALL and pre-B-ALL patients. The results indicate that the leukemia cells in B-ALL patients are composed of a large number of subclones, and the leukemia cell subpopulations in the B-ALL patients are heterogeneous.

### 3.2 Residual lymphoid cell subpopulations in B-all patients after induction therapy

The lymphoid cell subpopulations were measured in the BM samples from the B-ALL patients after the 1st course of induction therapy. The distribution of the residual lymphoid cell subpopulations was highly variable (Fig. 2) and was quite different from the distribution of the leukemia cell subpopulations in the patient samples obtained at diagnosis. Compared to the samples obtained at diagnosis, most of the lymphoid cell subpopulations were significantly decreased after induction therapy. In contrast, a fraction of the cell subpopulations maintained stable percentages, and some cell subpopulations even presented an increased percentage after induction therapy (Supplementary Table 1). The changes in the CLP-like populations in common ALL BCR/ABL^+^ patients after induction therapy were less than those in common ALL BCR/ABL^−^ patients. The changes in other stage subpopulations in the common ALL BCR/ABL^+^ patients and BCR/ABL^−^ patients were not significantly different (Table 3).

**Table 3:**
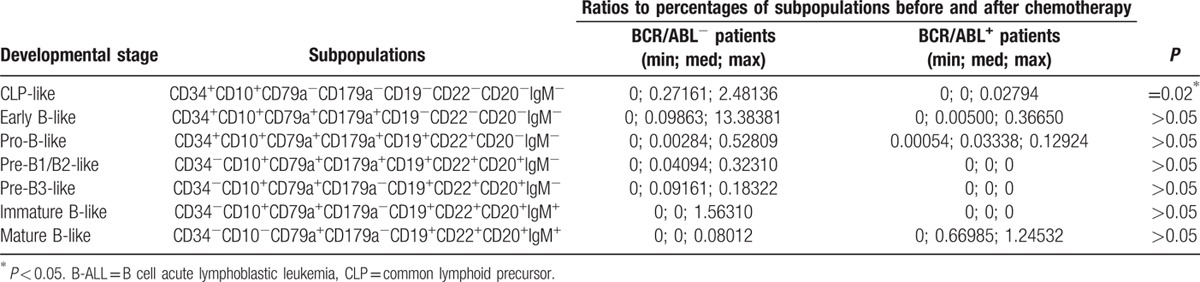
The ratios of the developmental stage-like B cells from common B-ALL patients before and after chemotherapy^[3,11]^ .

The residual CD34^+^CD10^−^ populations in the common ALL BCR/ABL^+^ patients were obviously reduced compared to those in the BCR/ABL^−^ patients after induction therapy (Fig. 4). However, the mean numbers of subpopulations with a stable or increased percentage were 10.1 subpopulations/patient in the BCR/ABL^+^ patients and 9.4 subpopulations/patient in the BCR/ABL^−^ patients. The mean numbers of subpopulations with a stable or increased percentage in the BCR/ABL^+^ and BCR/ABL^−^ common ALL patients were not significantly different. Of the CD10^+^ populations, the residual lymphoid cell subpopulations with a stable or increased percentage in common ALL patients with BCR/ABL^+^ were obviously reduced compared to those in the BCR/ABL^−^ patients (Fig. 4). In the CD34^+^CD10^+^CD19^−^ population with no or single expression of CD79a or CD179a, the numbers of cells in the CD20^−^ populations were increased compared to the numbers of cells in the CD20^+^ populations after induction therapy. Of the CD34^+^CD10^+^CD19^+^CD79a^+^CD179a^+^ population, the numbers of lymphoid cells in the common ALL BCR/ABL^+^ patients were increased compared to the CD34^+^CD10^+^CD19^−^CD79a^+^CD179a^+^ and CD34^+^CD10^+^CD19^+^ populations, with no or single expression of CD79 or CD179a but were less significant than those in the common ALL BCR/ABL^−^ patients. Of the CD34^−^CD10^+^CD19^+^CD179a^+^ population, the number of lymphoid cells in the common ALL BCR/ABL^+^ patients (mean: 5.7 subpopulations/patient) was reduced compared to the BCR/ABL^−^ patients (mean: 9.5 subpopulations/patient). Of the CD34^−^CD10^+^CD19^+^CD179a^−^ population, the number of lymphoid cells in the common ALL BCR/ABL^+^ patients (mean: 3.7 subpopulations/patient) was reduced compared to the common ALL BCR/ABL^−^ patients (mean: 8 subpopulations/patient). There was only 1 subpopulation with a stable or increased percentage in the 7 common ALL BCR/ABL^+^ patients, and the RPS was 1.04. Meanwhile, the 10 common ALL BCR/ABL^−^ patients had 24 subpopulations with a stable or increased percentage, and the RPS after chemotherapy exhibited a minimum value of 1.10, a median value of 3.74, and a maximum value of 26.38. These results indicate that the induction therapy had a stronger inhibitory effect against the CD10^+^ leukemia cell populations in the common ALL BCR/ABL^+^ patients than on those in the BCR/ABL^−^ patients. However, in the CD34^−^CD10^+^CD19^+^CD179a^+^ population, the number of subpopulations with a stable or increased percentage in the common ALL BCR/ABL^+^ patients was 0.7 subpopulations/patient, and the RPS of the subpopulations with a stable or increased percentage in the common ALL BCR/ABL^+^ patients after chemotherapy exhibited a minimum value of 1.01, a medium value of 1.62, and a maximum value of 2.79. Meanwhile, in the common ALL BCR/ABL^−^ patients, the number of subpopulations with a stable or increased percentage was 0.8 subpopulations/patient, and the RPS of subpopulations with a stable or increased percentage in the common ALL BCR/ABL^−^ patients after chemotherapy exhibited a minimum value of 1.23, a medium value of 2.48, and a maximum value of 9.12. There was no significant difference in the RPS values between the common ALL BCR/ABL^+^ and BCR/ABL^−^ patients (*P* > 0.05). In the CD34^−^CD10^−^CD19^+^ population, the numbers and RPS of the lymphoid cell subpopulations were not different between the common ALL BCR/ABL^+^ and BCR/ABL^−^ patients (Figs. 2 and 4).

There were 3 common ALL patients who were positive for genes other than BCR/ABL. In patient 10, who expressed TEL/AML1, most of the lymphoid cell subpopulations were decreased and the CD34^+^CD10^+^CD19^+^ population accumulated after induction therapy. In patient 11, a HOX11^+^patient, half of the lymphoid cell subpopulations were maintained and were primarily distributed into the CD34^−^CD10^−^CD19^+^ population. Compared to the samples obtained at diagnosis, the percentages of 15 subpopulations increased more than 5-fold after chemotherapy, the percentage of subpopulation exhibited the maximum 725-fold increase. Most of the subpopulations did not express CD20. One subpopulation was CD20^+^. In case 12, an E2A-PBX1^+^patient, most of the lymphoid cell subpopulations were evenly distributed throughout all of the populations that appeared during B cell development after induction therapy. The lymphoid cell subpopulations with a stable or increased percentage were mainly located in the CD34^+^CD10^−^ population that expressed 1 or 2 additional antigens (Figs. 2 and 4).

Of the CD34^+^CD10^−^ population, the values of the subpopulations with a stable or increased percentage were 5 and 0 in 2 pro-B-ALL patients, respectively, which were obviously reduced compared to the common ALL patients. Most of the residual subpopulations with a stable or increased percentage did not express CD20. In a pre-B-ALL patient, the residual subpopulations with a stable or increased percentage were primarily the CD34^+^CD10^−^ and CD34^−^CD10^−^CD19^+^ populations. The numbers of cells in the CD20^−^ populations were increased compared to the numbers of cells in the CD20^+^ populations. There were no residual lymphoid cell subpopulations with a stable or increased percentage in the CD10^+^ populations.

### 3.3 New lymphoid cell subpopulations were identified in 23 patients after induction therapy

After induction therapy, there were some new lymphoid cell subpopulations that had not been detected at diagnosis. The new subpopulations were observed in 22 patients, and the numbers and distributions of these new subpopulations varied (Fig. 3). An average of 10.4 subpopulations/patient (minimum: 0, maximum: 33 and median: 10) were observed in common ALL BCR/ABL^+^ patients, while an average of 20.6 subpopulations/patient (minimum: 2, maximum: 67 and median: 14) were observed in common ALL BCR/ABL^−^ patients, which significantly surpassed the numbers of the new subpopulations in the common ALL BCR/ABL^+^ patients (*P* < 0.05). The new subpopulations were primarily CD34^+^CD10^−^ and only expressed IgM, CD79a, and CD179a. Of the CD34^−^CD10^+^CD19^+^ population, an average of 0.9 subpopulations/patient (minimum: 0, maximum: 2 and median: 0) were observed in the common ALL BCR/ABL^+^ patients, while an average of 2.8 subpopulations/patient (minimum: 0, maximum: 12 and median: 1.5) were observed in the common ALL BCR/ABL^−^ patients, which was increased compared to the common ALL BCR/ABL^+^ patients (*P* < 0.05). In the CD34^−^CD10^−^CD19^+^ population, there were only 7 new subpopulations identified in common ALL BCR/ABL^+^ patients. These results indicate that the new subpopulations were primarily distributed throughout the CD34^+^CD10^−^ and CD34^−^CD10^−^CD19^+^ populations after induction therapy. The common ALL BCR/ABL^−^ patients exhibited a greater ability to generate new subpopulations, suggesting that the common ALL BCR/ABL^−^ patients have exhibited more clonal evolution after induction therapy (Fig. 3).

In common ALL patients who expressed TEL/AML1^+^, the 11 new subpopulations were present at a high frequency in the CD34^−^CD10^+^CD19^+^CD179a^+^ and CD34^−^CD10^−^CD19^+^ populations, which were higher than those in the other patients. There were only 4 new subpopulations in the CD34^+^CD10^+^CD19^−^ population with no or single expression of CD79a or CD179a, 10 new subpopulations in the CD34^+^CD10^+^CD19^−^CD79a^+^CD179a^+^ and CD34^+^CD10^+^CD19^+^ populations with no or single expression of CD79 or CD179a, 3 new subpopulations in the CD34^+^CD10^+^CD19^+^CD79a^+^CD179a^+^ population, and 11 new subpopulations in the CD34^−^CD10^+^CD19^+^CD179a^+^ population. Twenty-eight new subpopulations appeared in the common ALL patient who expressed E2A-PBX1. Half of them were CD34^+^CD10^−^. In addition, 1 and 2 new subpopulations were identified in the 2 pro-B-ALL patients, respectively, and 4 new subpopulations were identified in the pre-B-ALL patient (Fig. 3).

**Figure 5:**
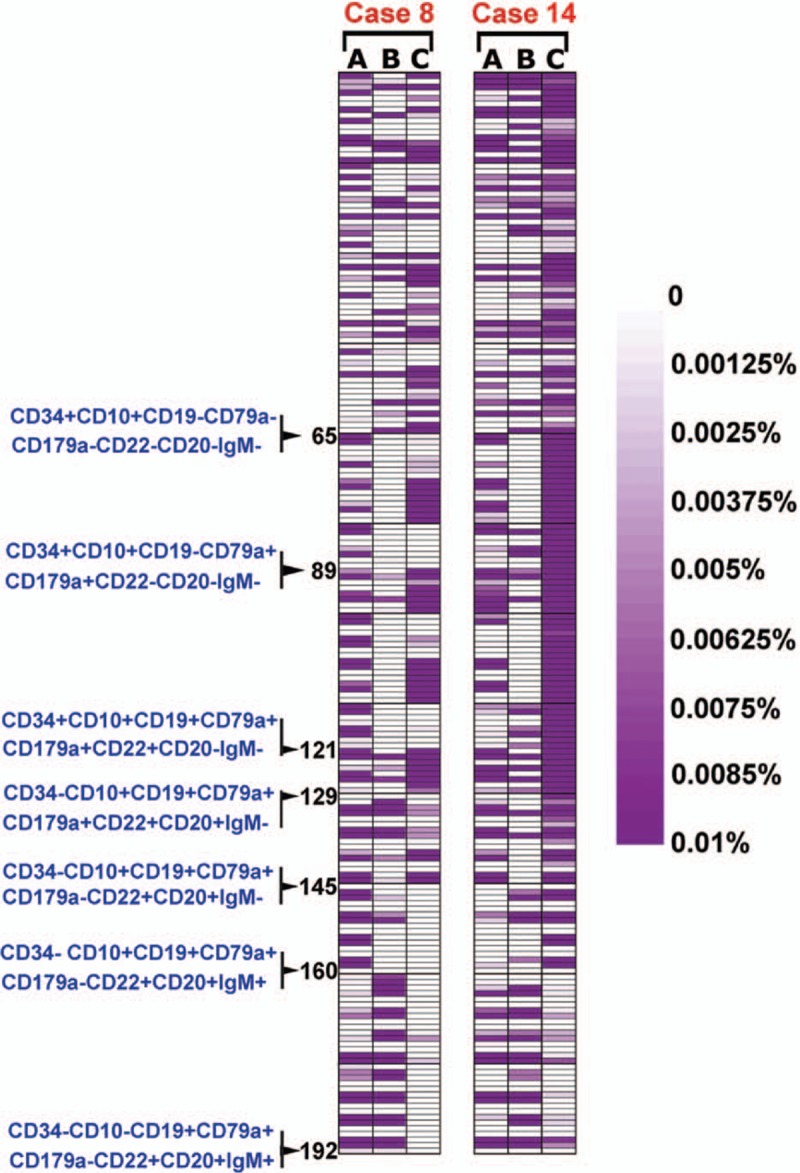
The changes in the subpopulations at diagnosis, after induction therapy and at relapse in 2 relapsed case. The figure represents the changes in the subpopulations at diagnosis (A), after induction therapy (B), and at relapse (C) in 2 relapsed case. The range of 0% to 0.01% (0–100 cells) was defined, and with increasing numbers of cells, the purple color deepened more and reaching the maximum of 0.01% (100 cells), where the purple was the deepest.

Two patients (patient 8 and 14) relapsed after induction therapy. The distribution of leukemia cell subpopulations at relapse was significantly different from the patterns at diagnosis (Fig. 5). Patient 8, who was a common ALL BCR/ABL^+^ patient, relapsed at 13 weeks after induction therapy. After induction therapy, the residual cell subpopulations were mainly located in the CD34^+^CD10^−^, CD34^−^CD10^+^CD19^+^CD179a^+^, and CD34^−^CD10^−^CD19^+^ populations. The number of subpopulations with a stable or increased percentage was 26 subpopulations. At relapse, the lymphoid cell subpopulations that had been suppressed by the induction therapy rebounded, with a large number of cells in the CD34^+^CD10^+^CD19^−^ and CD34^+^CD10^+^CD19^+^ populations with no or single expression of CD79 or CD179a. The subpopulations that expressed CD179a exhibited a stable or increased percentage. Patient 14, a common ALL BCR/ABL^−^ patient, relapsed at 14 weeks after induction therapy. The lymphoid cell subpopulations were mainly located in the CD34^+^CD10^−^, CD34^+^CD10^+^CD19^−^, CD34^−^CD10^−^CD19^+^, and CD34^+^CD10^+^CD19^+^ populations with no or single expression of CD79 or CD179a, and 14 subpopulations exhibited a stable or increased percentage. At relapse, the lymphoid cell subpopulations in the CD34^+^CD10^+^CD19^−^ and CD34^+^CD10^+^CD19^+^ populations with no or single expression of CD79 or CD179a that had been suppressed by the induction therapy had recovered. The lymphoid cell subpopulations with a stable or increased percentage persisted. Thirty-eight subpopulations presented a higher percentage. Interestingly, the subpopulations of the CD34^−^CD10^−^CD19^+^ population accumulated in 2 relapsed patients after induction therapy. The subpopulations in the CD34^−^CD10^−^CD19^+^ population significantly declined at relapse. These results suggest that the main lymphoid cell subpopulations in the CD34^+^CD10^+^ population that existed at diagnosis recovered at relapse, although they were rapidly and substantially decreased after induction therapy. The subpopulations with a stable or increased percentage after induction therapy persistently increased at relapse and were considered to be the main component of the lymphoid cells at relapse. The residual lymphoid cell subpopulations in the CD34^−^CD10^−^CD19^+^ population dramatically decreased at relapse following induction therapy. Of the 2 relapsed patients, new subpopulations were also detected at relapse that never existed at diagnosis and after induction therapy. The new subpopulations were mainly observed in the CD34^+^CD10^−^ populations.

## 4 Discussion

Currently, PCR and FCM are used to evaluate the treatment response of patients with B-ALL. PCR is mainly used to detect clonal rearrangements of immunoglobulin (Ig) genes or fusion genes. However, PCR assays of IgH genes are labor-intensive, time-consuming, highly complex, and expensive.^[24–26]^ Real-time quantitative PCR is also used to detect fusion genes, such as BCR/ABL1, MLL/AFF1, TCF3/PBX1, and ETV6/RUNX1. Real-time quantitative PCR is simple, fast, and specific, with sensitivities ranging from 10^−3^ to 10^−5^, based on the different types of fusion genes. However, currently, fusion genes only can be detected in less than 1/3 of ALL patients.^[25]^ Leukemia-associated immunophenotypes are most commonly used to detect minimal residual disease by FCM. Compared to PCR, FCM is much faster and relatively simple. In addition, FCM has the advantage of single cell analysis and is able to recognize and characterize small subpopulations and exclude dead cells. Compared with classic FCM, MFC can dramatically conserve samples and reagents and also offer more accuracy in identifying cell populations.^[23,27]^ Recently, MFC has been increasingly used to manage B-ALL, with high sensitivity and specificity, and has been regarded as an important counterpart of PCR detection.^[23,25,28,29]^ B cell development is associated with the oncogenesis, treatment, and prognosis of B-ALL.^[1,17,30]^ In our experiments, we used a panel based on B cell development that is able to detect the tumor cell subpopulations in B-ALL patients and define and analyze the 7 B cell developmental stages by examining the cytoplasmic antigens CD179a and CD79a. Although 8-color panels using some antigens that are associated with B cell development have been applied to detect the leukemia cell populations in B-ALL, the panels are incomplete and cannot perform a comprehensive analysis of all stages of B cell development in one tube; they are only able to obtain some of the information that has been correlated with the asynchronous expression of B cells.^[16,23]^ In contrast, our panel is able to detect all of the normal B cells that appeared during development in one tube. Therefore, the panel is able to examine all B-ALL specimens. Currently, Sequenta LymphoSIGHT sequence analysis is applied to examine the samples of B-ALL patients after chemotherapy and is able to perform a quantitative and sensitive analysis. The sensitivity of the sequence assay is also limited by the number of input cells and shows higher sensitivity than the standard ASO-PCR- and FCM-based methods, if the number of input cells is appropriate.^[29]^ However, the sequence analysis is more expensive, is highly dependent on the equipment, and cannot be broadly applied. The sensitivity of MFC is dependent on acquiring a large number of cells.^[31]^ In our experiments, 5 × 10^5^ cells in each specimen were acquired for the MFC examination. The results of these examinations show a similar sensitivity as PCR.

B-ALL is a heterogeneous disease, characterized by various clinical features and different cancer cells.^[32,33]^ Childhood B-ALL often displays significant heterogeneity in its morphology, immunophenotype, genetic aberrations, and response to therapy.^[34]^ Gawad et al^[4]^ observed clonal IgH rearrangements in 43 of 51 childhood B-ALL patients at diagnosis, and the number of evolved IgH sequences per patient ranges dramatically from 0 to 4024. In a BCR/ABL1^+^ B-ALL patient, the “Stem/B” cells selectively persisted at remission, whereas no BCR/ABL1^+^ lymphoid cells could be observed in the other B-cell populations. “Stem/B” cells are more quiescent and less actively cycling than leukemic “Pro-B” cells, highlighting the importance and feasibility of tracking rare but distinct leukemia cell populations in patients undergoing therapy.^[19]^ Obro et al^[3]^ reported that the subpopulations are most commonly characterized by a bimodal expression of CD34 in childhood B-ALL patients. All of the flow-sorted presumed leukemia cell populations show a similar dominance of the cytogenetically aberrant cells, with 60% to 100% FISH-positive cells, irrespective of the bimodal or broad expression of the defining marker. We have also found that adult common ALL patients are highly heterogeneous and carry distinct subpopulations that are defined by bimodal antigen expression in more than half of the patients.^[5]^ These data provide clues for individualized therapies and therapeutic monitoring in B-ALL patients. In our present study, the panel is able to elucidate the stages of B cell development in one tube. The results show a significant heterogeneity of the leukemia cell subpopulations in the B-ALL patients. Meanwhile, the responses of all lymphoid cell subpopulations in the patients to the 1st course of induction therapy were also variable. For most lymphoid cell subpopulations, the numbers and percentages decreased in patients following induction therapy. There are still many lymphoid cell subpopulations with a stable or increased percentage. Although we have not sorted these residual subpopulations to assay clonality, many residual subpopulations with asynchronous antigens are observed, such as the coexpression of CD34^+^CD20^+^ and CD34^+^IgM^+^, which do not exist during normal B cell development and are regarded as residual B-ALL subpopulations. The poor prognostic impact of Ph^+^ ALL is well known. Patients with Ph^+^ ALL are less likely to get CR and exhibit CR rates of approximately 70% to 80%.^[33]^ Our results indicated that the therapy has a stronger effect in the common ALL BCR/ABL^+^ patients who received TKI than that in the BCR/ABL^−^ patients who display the CD10^+^ population, while there were no differences in the other populations. These results indicate that the population containing the CD10^+^ subpopulations is sensitive to Imatinib and is rapidly cleared in the adult BCR/ABL^+^ patients. Therefore, our results demonstrate that Imatinib is able to selectively eliminate certain B-ALL subpopulations. The underlying mechanism requires additional study.

In our study, the 2 relapsed patients carry many residual CD34^+^CD10^−^ and CD34^−^CD10^−^CD19^+^ subpopulations after induction therapy. However, at relapse, the CD34^−^CD10^−^CD19^+^ subpopulations are obviously decreased. The CD34^+^CD10^−^ subpopulations are stable or are increased. This suggests that the residual subpopulations may consist of regenerated normal CD34^−^CD10^−^CD19^+^ B cells following induction therapy, whereas the residual subpopulations may consist of CD34^+^CD10^−^ ALL cells following induction therapy. The mechanisms are still unclear.

Generally, cancer results from the accumulation of multiple mutations in a single cell lineage that are sequentially acquired and subject to an evolutionary process where selection drives the expansion of more fit subclones. However, many aspects of clonal evolution are poorly understood.^[35–37]^ Evidence for clonal evolution in B-ALL had been reported.^[38–40]^ The morphology and immunophenotype from 2 cases of B-ALL have definitely changed at relapse compared to that at diagnosis and after induction and consolidation therapy.^[38]^ Our data show that new subpopulations are identified in 22 patients. Many subpopulations exhibit asynchronous expression and suggest that, in most patients, clonal evolution can occur after induction therapy. Moreover, the subpopulations of clonal evolution are primarily in CD34^+^CD10^−^ and CD34^−^CD10^−^CD19^+^ population. There are few subpopulations in the CD34^−^CD10^−^CD19^+^ population, which is not the main component of the subpopulations present in the 2 patients who relapsed. The number of new subpopulations in common ALL BCR/ABL^−^ patients is higher than that in the patients with BCR/ABL^+^. In addition, the common ALL BCR/ABL^−^ patients exhibit stronger clonal evolution compared to the BCR/ABL^+^ patients. Sequenta LymphoSIGHT can define clonal evolution. The method is actually an optimized and high throughput PCR-based method to detect IgH rearrangements.^[29]^ In our study, MFC, which is based on B cell development, is able to detect the subpopulations of evolved clones, detect more of the small subpopulations, and gain a deeper recognition of clonal evolution in ALL patients.

In summary, we observed the heterogeneity of B-ALL subpopulations using MFC based on the development of B lymphocytes and demonstrated that adult B-ALL patients often have a massive collection of subtly divergent leukemic subclones. The responses of the different subpopulations to the 1st course of induction therapy were also heterogeneous. Subpopulations of clonal evolution were heterogeneous after induction therapy. Our observations suggest that the subpopulations in B-ALL patients should be dynamically monitored to analyze the effect of therapies and to predict the prognosis of the disease.

## Acknowledgments

The authors thank the participants for their kind cooperation, generosity, and patience. The authors also thank National Basic Research Program of China (to XM, 2015CB942800) and the Natural Science Foundation of China (to HX 31171384, to XM 81361120381) for the support.

## Supplementary Material

Supplemental Digital Content
