# Supplementary material for: Development-associated immunophenotypes reveal the heterogeneous and individualized early responses of adult B-acute lymphoblastic leukemia
Source: Medicine (Baltimore). 2016 Aug 26;95(34):e4128. doi: 10.1097/MD.0000000000004128 (PMC5400307; doi:10.1097/MD.0000000000004128)
Supplement: Supplemental Digital Content [file medi-95-e4128-s001.pdf]

Supplemental Figure 1 legend

1a

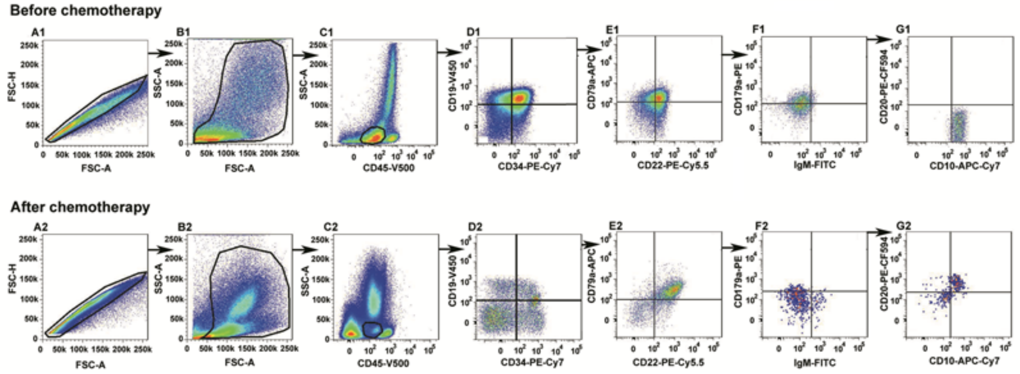

1b

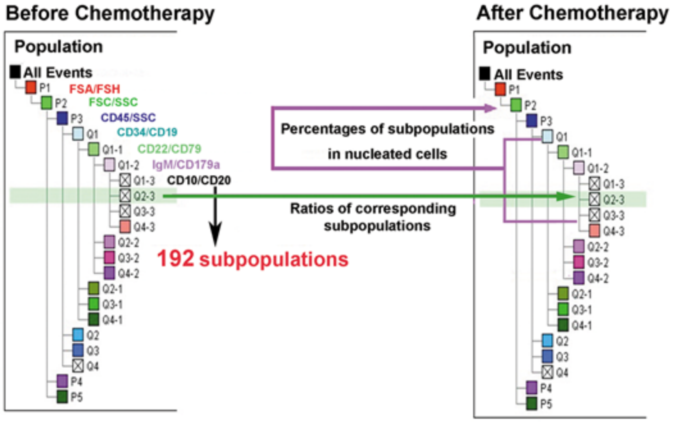

1a, the aggregated cells were excluded from the forward scatter (FSC) height versus FSC area dot plots. The dead cells and debris were excluded from the FSC/side scatter (SSC) dot plots. The blast populations were identified using CD34/CD19 dot plots after being analyzed by the SSC/CD45 dot plots. The $CD34^{-}CD19^{-}$ population was excluded because it contained cell debris and erythroblasts; the other 3 main populations were gated further. **1b**, the percentage of subpopulation was obtained by dividing the number of nucleated cells by the number of cells in the subpopulation. To compare the percentage of the subpopulation among the same immunophenotypes in different B-ALL patients at diagnosis, after induction chemotherapy and at relapse, the changes in the percentages of the corresponding subpopulations were obtained. They can be used to characterize the changes in the leukemia cell subpopulations as stable, increased or decreased at diagnosis and after induction therapy.

There are many other kinds of medicines used in addition to chemotherapy drugs.

Treatment	Category	Generic drug name
Support treatment	Blood Components	Red cells / Plasma
	Nutrition and fluid	NS / vitamin C / Potassium, Magnesium, Calcium and glucose injection/ Mannatide/Mecobalamin, etc.
Anti-infection treatment	Antibiotics	Tienam / Cravit / Amikacin / Piperacillin and sulbactum / Methicillin-resistant Staphylococcus / Metronidazole etc.
	Antifungus	Voriconazole / Fluconazole / Amphotéricine B
	Anti-virus	Acyclovir
Symptomatic treatment	Hemostasis therapy	Platelets / Carbazochrome / Tranexamic acid / Fibrinogen
	Pain Relief therapy	Indometacin / Dezocine
	White cells Reducing therapy	Hydroxyurea
	Granulocytopenia	rhGM-CSF
	others	Insulin; Polyene phosphatidyl choline / Tiopronin; Allopurinolurate, etc.
